# Understanding HLA associations from SNP summary association statistics

**DOI:** 10.1038/s41598-018-37840-9

**Published:** 2019-02-04

**Authors:** Jiwoo Lim, Sang-Cheol Bae, Kwangwoo Kim

**Affiliations:** 1 Department of Biology, Kyung Hee University, Seoul, Republic of Korea; 2 Department of Rheumatology, Hanyang University Hospital for Rheumatic Diseases, Seoul, Republic of Korea

## Abstract

Strong genetic associations in the region containing human leukocyte antigen (HLA) genes have been well-documented in various human immune disorders. Imputation methods to infer HLA variants from single nucleotide polymorphism (SNP) genotypes are currently used to understand HLA associations with a trait of interest. However, it is challenging for some researchers to obtain individual-level SNP genotype data or reference haplotype data. In this study, we developed and evaluated a new method, DISH (direct imputing summary association statistics of HLA variants), for imputing summary association statistics of HLA variants from SNP summary association statistics based on linkage disequilibria in Asian and European populations. Disease association Z scores in DISH were highly correlated with those from imputed HLA genotypes in null model datasets (r = 0.934 in Asians; r = 0.960 in Europeans). We applied DISH to two previous GWAS datasets in Asian systemic lupus erythematosus and European rheumatoid arthritis populations. There was a high correlation between Z scores in the DISH and HLA genotype imputations, showing the same disease-susceptible and protective alleles. This study illustrated the usefulness of the DISH method in understanding and identifying disease-associated HLA variants in human diseases while maintaining individual-level data security.

## Introduction

Human leukocyte antigens (HLAs) present short peptides of self or foreign antigens on the cell surface to T lymphocytes^1^. HLA genes are highly polymorphic. According to the IPD-IMGT/HLA database^2^ (Release 3.34.0 on Oct-18 2018), 8 major HLA genes (*HLA-A*, -*B*, -*C*, -*DRB1*, -*DPA1*, -*DPB1*, -*DQA1*, and -*DQB1*) have >16,000 HLA alleles, encoding for more than 13,000 protein variants.

Genetic associations within the major histocompatibility complex (MHC) region, which contains HLA genes, has been well characterized in various human inflammatory disorders, such as rheumatoid arthritis (RA) and systemic lupus erythematosus (SLE)^3,4^. To further understand the genetic architecture and functional variants in HLA disease association, HLA imputation has been widely used to infer individual HLA classical alleles and amino acid residues from single nucleotide polymorphism (SNP) genotypes using a hidden Markov model (HMM)-based imputation method^5,6^. However, individual-level SNP data to impute HLA alleles are typically limited or difficult for other researchers to access because of potential ethical concerns regarding sharing individual data and data security. Moreover, reference panels to provide long-range haplotypes constructed from HLA and SNP variants are not always available publicly. For example, a large European HLA reference panel (T1DGC panel) was initially provided with the SNP2HLA program^5^, but is no longer available in order to protect personal genetic information.

In contrast to the individual-level GWAS data, sharing summary association statistics, including effect sizes, standard errors, Z-scores, and *P* values for SNPs, has become more prevalent in recent years due to voluntary sharing and journal policies. Herein, we introduce a computational tool to impute HLA summary association statistics from SNP summary association statistics (without individual-level SNP data and individual-level reference panel data) using linkage disequilibrium (LD) information between SNP and HLA variants in European and Asian populations. This method uses existing statistical techniques to impute the associations of untyped SNPs with a trait of interest based on the summary association statistics of typed SNPs. We illustrate that imputation of HLA association statistics can help understand causal HLA alleles hidden in nearby SNP association signals.

## Methods

### Overview of the method to impute summary association statistics of HLA variants

Local allelic correlations of genetic variants cause correlations of disease association statistics including Z scores of the same variants. Z scores can be approximated by multivariate normal (MVN) distribution *N*(0,*V*) where the variance *V* is variance-covariance/correlation matrix and is equal to the LD correlation (*r*) matrix. Based on the conditional expectation of MVN variates, several studies have provided programs to impute Z scores of untyped SNP from Z scores of typed SNPs and reference LD information^7–10^. In this study, we applied the same mathematical approach to impute HLA association statistics. Z scores in a given locus are partitioned into Z scores of typed SNPs (*Z*_*SNP*_) and untyped HLA variants (*Z*_*HLA*_). The conditional expectation of *Z*_*HLA*_ given *Z*_*SNP*_ is estimated by

$$E({Z}_{HLA}|{Z}_{SNP})={{\rm{\Sigma }}}_{HLA,SNP}\,{{\rm{\Sigma }}}_{SNP,SNP}^{-1}\,{Z}_{SNP}$$

where ${{\rm{\Sigma }}}_{HLA,SNP}$ is the covariance matrix among HLA variants and SNPs and ${{\rm{\Sigma }}}_{SNP,SNP}$ is the covariance matrix among SNPs. To adjust for statistical noise and ensure that the covariance matrix is invertible, ${{\rm{\Sigma }}}_{SNP,SNP}$ is adjusted by adding a value (λ) at the diagonal element of the matrix (default λ = 0.15; Supplementary Fig. 1, Table 1, and Table 2). These two covariance matrices are calculated from well-validated Asian and European reference datasets that contain long-range haplotypes consisting of SNP and HLA variants (including HLA classical alleles and amino acid residues) within the MHC region in 5,225 European or 854 Asians. Specifically, the European reference dataset was generated by the Beagle program to phase binary codes of 5,868 SNPs, 126 one-field and 298 two-field HLA alleles (for *HLA-A*, *-B*, *-C*, -*DPA1*, -*DPB1*, -*DQA1*, -*DQB1* and -*DRB1*) and 399 polymorphic amino acid positions into 10,450 haplotypes in 5,225 unrelated Europeans^5^. The Asian reference dataset was phased by the same method to include 4,758 SNPs, 86 one-field and 163 two-field HLA alleles (for *HLA-A*, -*B*, -*C*, -*DPA1*, -*DPB1*, -*DQA1*, -*DQB1* and -*DRB1*) and 1,528 polymorphic amino acid position into 1,708 haplotypes in 854 unrelated Asians. All the two-field HLA alleles were obtain by high-resolution sequence-based HLA typing^5,11,12^. The reference datasets were used as reference panels in imputing individual-level genotypes of HLA variants. The more detailed results of the reference datasets are described elsewhere^5,13^. Our computational strategy, DISH (direct imputing summary association statistics of HLA variants) is implemented in R and is publicly available at https://github.com/Ben-JWL/DiSH. We note that individual-level data of both the European and Asian references are not publicly available at this time. Instead, we precomputed and provided covariance matrices to protect individual genetic information and to make imputations run faster. The imputation reliability at each HLA variant was assessed by *r*^2^*pred*, the variance of the conditional variable ${Z}_{HLA}|\,{Z}_{SNP}$, as previously described^10^.

### Application of the Z-score imputation method to RA and SLE datasets

We generated two null-model datasets from pre-existing European and Asian genome-wide association studies (GWAS) data, respectively. European GWAS data was obtained from the Wellcome Trust Case-Control Consortium (WTCCC) (dataset accession ID: EGAD00000000021 and EGAD00000000022). A total of 2,962 WTCCC2 controls from the 1958 British Birth Cohort were genotyped by both Illumina 1.2 M array and Affymetrix_6.0 array. Data were merged and processed by general quality control (QC) procedures. The Asian SLE GWAS data was obtained from our previous study. In brief, a total of 5,342 unrelated QC-passed Korean subjects, including 849 SLE cases and 4,493 controls, were genotyped by Illumina Omni1 arrays and Human610/660W-Quad arrays. The 10,000 null-model datasets were generated by randomly assigning phenotypes (1000 cases and 1000 controls in each dataset) to samples from the original GWAS data. The null-model datasets were used to calculate Z scores of each SNP and to impute Z cores of untyped variants by DISH using an ethnicity-matched reference LD matrix. The accuracy of imputed Z scores in the DISH method was evaluated by comparing with Z scores calculated from imputed individual genotypes that were generated by HMM-based genotype imputation using SNP2HLA.

In addition, we applied our method to previously reported GWAS summary association statistic data and individual-level data from rheumatoid arthritis in Europeans^14^ and systemic lupus erythematosus in Asians^13^, respectively. For the European dataset, only association statistics for SNP2HLA-imputed variants was publicly available. From the statistics summary, we arbitrarily created a DISH input consisting of only the biallelic SNPs with official SNP names. For the Asian dataset, we calculated actual Z scores from genotyped data in order to impute DISH Z scores of untyped HLA variants using DISH-based methods.

## Results

### Performance of GWAS data with random disease phenotypes

We designed the DISH method to impute *Z* scores for genetic associations of untyped SNP and HLA variants from typed SNPs in a 5-Mb window of the MHC region based on a single variance-covariance matrix of the total variants listed in the reference dataset (see Details in Methods). To evaluate the performance of HLA statistic imputation, we generated 10,000 null model datasets from previously reported Asian^13^ or European (WTCCC2) GWAS data by randomly selecting 1,000 disease-affected cases and 1,000 controls and obtained Z scores of untyped variants by HMM-based and DISH-based imputation. The number of overlapping SNPs between the GWAS data and the ethnicity-matched reference variance-covariance matrix was 3,073 SNPs in Europeans and 2,582 SNPs in Asians. The HMM-based Z scores were obtained by testing for disease associations with SNP2HLA-imputed dosages of untyped variants. The DISH-based Z scores of untyped variants for disease association were calculated from Z scores of typed variants. The correlation coefficient (*r*) between the two independently generated sets of Z scores with an *r*^2^*pred* value ≥ 0.5 was 0.934 in the Asian dataset and 0.960 in the European dataset (Supplementary Fig. 1). Under the setting parameter (λ = 0.15), there appears to be 0.70 to 0.79-fold fewer associated variants from DISH-based Z scores at mild significance thresholds (*P* at 0.05 to 5 × 10^−4^) compared to HMM-based Z scores. However, this deflation is critical to control the type I error in SNPs with strong *P* values (Supplementary Tables 1 and 2).

### Performance with previous datasets

**Figure 1:**
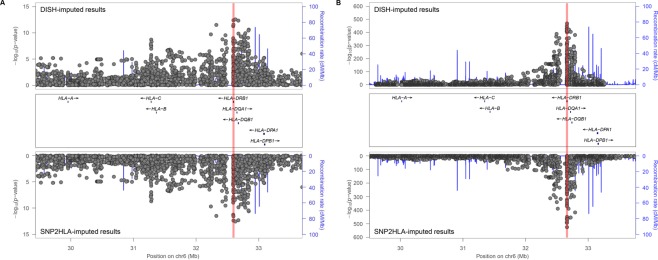
Significance levels of variants in the HLA region calculated from disease association Z scores in DISH and SNP2HLA were transformed in the –log_10_ scale and plotted for (**A**) systemic lupus erythematosus and (**B**) rheumatoid arthritis according to chromosomal positions. Results from DISH-based Z imputation and SNP2HLA HMM-based genotype imputation are shown in the upper and lower panels, respectively. The *HLA-DRB1* region is highlighted in red.

**Figure 2:**
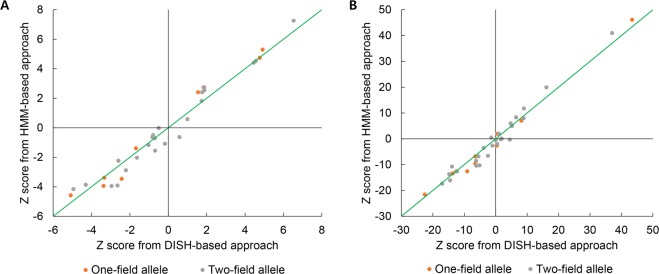
One-field and two-field classical alleles of *HLA-DRB1* were assessed for associations with systemic lupus erythematosus (SLE) and rheumatoid arthritis (RA) using DISH-based Z imputation and SNP2HLA HMM-based genotype imputation. Scatter plots for (**A**) SLE and (**B**) RA show the correlation between disease association Z scores in DISH and SNP2HLA. One-field and two-field alleles are shown as gray and orange, respectively. The green diagonal line indicates the same Z score values.

To illustrate the advantage of DISH in identifying HLA variants associated with diseases, we applied our method to two previous GWAS datasets in Asian SLE and European RA populations. Both diseases were most significantly associated with *HLA-DRB1* variants in DISH and HMM-based SNP2HLA imputations (Fig. 1). For the Asian SLE GWAS data, DISH-imputed and HMM-imputed Z scores were highly correlated (*r* = 0.962 for markers with *r*^2^*pred* value ≥ 0.6). The two-field alleles of *HLA-DRB1* showed similar association significance levels using DISH-based and HMM-based strategies. The Z score ranking of *HLA-DRB1* alleles was also highly consistent (Spearman’s rank correlation coefficient *r* = 0.975; Fig. 2A). In both methods, HLA-DRB*15:01 showed the most significant risk association (*Z*_DISH_ = 6.53, *r2ped* = 0.92, *P*_DISH_ = 6.37 × 10^−11^; *Z*_SNP2HLA_ = 7.26, *P*_SNP2HLA_ = 3.89 × 10^−13^), while HLA-DRB*04:01 showed the most significant protective association (*Z*_DISH_ = −5.09, *r2ped* = 0.94, *P*_DISH_ = 3.66 × 10^−7^; *Z*_SNP2HLA_ = −4.57, *P*_SNP2HLA_ = 4.79 × 10^−6^). Similarly, RA association of *HLA-DRB1* alleles displayed good correlation between both methods (Spearman’s rank correlation coefficient *r* = 0.974) with the riskiest allele HLA-DRB*04:01 (*Z*_DISH_ = 37.04, *r2ped* = 0.79, *P*_DISH_ = 2.44 × 10^−300^; *Z*_SNP2HLA_ = 40.99, *P*_SNP2HLA_ = 3.03 × 10^−367^) and the most protective allele HLA-DRB*13:01 (*Z*_DISH_ = −17.03, *r2ped* = 0.66, *P*_DISH_ = 5.24 × 10^−65^; *Z*_SNP2HLA_ = −17.38, *P*_SNP2HLA_ = 1.21 × 10^−67^; Fig. 2B).

## Discussion

In most cases, it is difficult to interpret the biological meaning of SNP associations in the MHC region because of few GWAS variants in the HLA coding variants and numerous trait-associated SNPs due to high LD in the MHC region. In this study, the DISH method was developed to impute association statistics of untyped HLA variants directly from the statistics of typed SNPs. This method works based on the LD information in covariance matrices from European or Asian reference samples without individual-level GWAS genotype and reference data. Several methods to infer Z scores of untyped variants have been reported in recent years^7–10^. In these methods, individual-level dense genetic information in various populations in the 1000 Genome Project was used to estimate LD structure. By contrast, DISH has two pre-calculated LD matrices for variants in the MHC region in the large T1DGC samples originally used in SNP2HLA imputation^5^ and for variants in a large Asian reference panel previously customized for an SLE-HLA association study^13^ in order to maintain confidentiality of all the personal genetic information and run a faster imputation. Using null GWAS datasets with European or Asian LD architecture and actual GWAS datasets (or association summary statistics) for European RA and Asian SLE populations, we showed that MVN-based DISH-imputed Z scores calculated from individual-level genotypes of typed variants were highly correlated with HMM-based Z scores calculated from individual-level imputed genotypes by SNP2HLA. Moreover, the most protective, susceptible alleles were identical in both methods, illustrating the usefulness of the DISH method in looking for the most significant alleles in HLA fine-mapping studies. The observed associations of *HLA-DRB1* alleles with RA or SLE in DISH-based and HMM-based approaches were supported by previous studies^15,16^ that tested for the association for sequenced *HLA-DRB1* alleles.

This method provided *Z* scores, *r2pred*, and *P* values of HLA and SNP variants listed only in the reference data. The HLA association summary statistics are generated for one-field and two-field alleles and all polymorphic amino acid residues in *HLA-A*, *-B*, *-C*, -*DPA1*, -*DPB1*, -*DQA1*, -*DQB1* and -*DRB1*. Thus, the trait-associated classical HLA alleles of the major HLA genes could be identified and the important amino-acid residues or positions could be further narrowed down from DISH outputs. However, we note that it is also possible not to detect a true primary association of HLA genes in DISH outputs especially when minor HLA genes (e.g., *HLA-DRB3*, *HLA-G*; not tested by DISH) or synonymous/intronic/UTR variants in the tested HLA genes are the actual primary causes. Furthermore, extensive and high LD patterns in the MHC region may interfere with detecting the true association signals especially for underpowered GWAS data before and after DISH-based imputation.

There are three major limitations to the DISH approach due to no individual-level data, similar to other association statistic-imputing programs. First, HLA genes are highly polymorphic at the amino acid or gene levels. However, significance levels for disease association can be calculated only for bi-allelic variants (e.g., SNP encoded as allele 1 and allele 2, HLA classical allele encoded as present and absent). The association statistics for multi-allelic variants (for example, from log-likelihood ratio tests or ANOVA,) are not able to be imputed from SNP association results and LD information. Second, it is not possible to correct for confounding factors (e.g., genetic background and environmental factors) and perform a conditional analysis that adjusts for the most significant trait-associated variant to identify another independently associated variant. There is a different statistic-imputing program (DISSCO) correcting for confounding covariates but requiring individual-level covariates^8^. Third, although the order of significant SNPs sorted by P value was highly correlated between DISH and SNP2HLA methods, actual *P* values could be substantially different. Small differences in Z scores may show as large differences in the logarithmically scaled *P* values.

In summary, we introduced the publicly available DISH method to impute HLA associations directly from summary association statistics of nearby SNP associations. This method is useful for understanding and identifying disease-associated HLA variants in human disease while maintaining individual-level data security.

## Supplementary information

Supplementary Information
