# Supplementary material for: Understanding HLA associations from SNP summary association statistics
Source: Sci Rep. 2019 Feb 4;9:1337. doi: 10.1038/s41598-018-37840-9 (PMC6362191; doi:10.1038/s41598-018-37840-9)
Supplement: Supplementary file 1 — Supplementary Information [file 41598_2018_37840_MOESM1_ESM.pdf]

Supplementary Information

Understanding HLA associations from SNP summary association statistics

Jiwoo Lim[1], Sang-Cheol Bae[2], and Kwangwoo Kim*[,1]

[1]Department of Biology, Kyung Hee University, Seoul, Republic of Korea.

[2]Department of Rheumatology, Hanyang University Hospital for Rheumatic Diseases, Seoul, Republic of Korea.

Correspondence and requests for materials should be addressed to KK (email: kkim@khu.ac.kr)

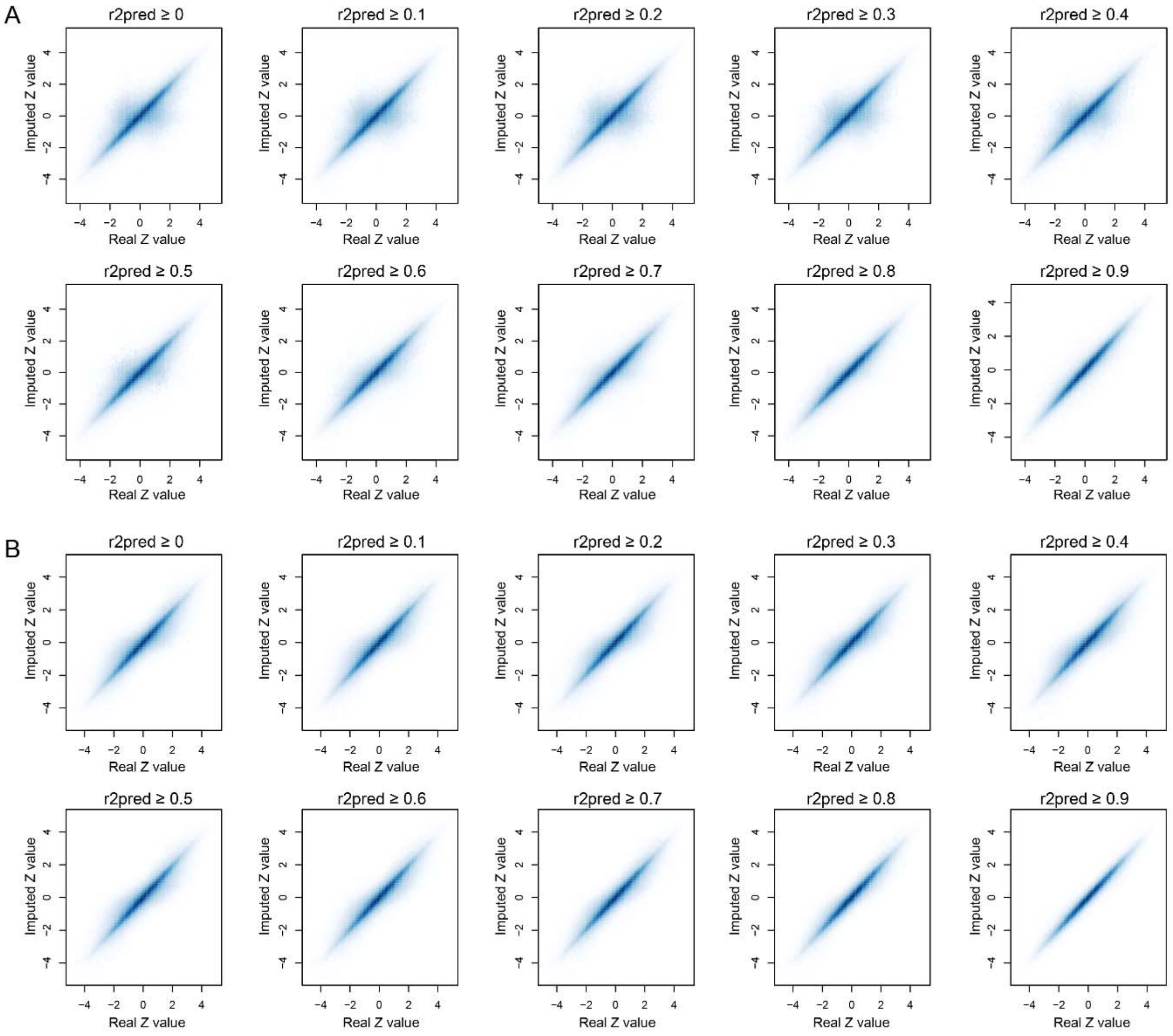

Supplementary Figure 1 | For GWAS data from (A) Asians and (B) Europeans with random disease phenotypes, DISH-imputed Z scores (y-axis) were plotted with Z scores from SNP2HLA-imputed genotypes at various *r2pred* thresholds (from 0 to 0.9).

Supplementary Table 1| Ratio of the observed number of significant associations in DISH results to SNP2HLA results at various lambda values, association *P* threshold levels, and *r2pred* values in the Asian null-model dataset.

	Association *P* threshold				
	0.5	0.05	0.005	0.0005	0.00005
	r2pred>=0				
λ=0.05	0.888	0.837	0.881	0.993	1.209
λ=0.10	0.865	0.784	0.802	0.861	1.093
λ=0.15	0.850	0.751	0.747	0.786	0.959
λ=0.20	0.839	0.724	0.699	0.722	0.866
λ=0.25	0.829	0.701	0.659	0.676	0.808
λ=0.30	0.821	0.682	0.623	0.640	0.756
	r2pred>=0.5				
λ=0.05	0.893	0.840	0.883	0.993	1.209
λ=0.10	0.896	0.806	0.808	0.861	1.093
λ=0.15	0.886	0.773	0.752	0.786	0.959
λ=0.20	0.878	0.747	0.705	0.723	0.866
λ=0.25	0.871	0.725	0.665	0.677	0.808
λ=0.30	0.896	0.708	0.629	0.641	0.756
	r2pred>=0.6				
λ=0.05	0.912	0.856	0.887	0.994	1.209
λ=0.10	0.898	0.809	0.810	0.863	1.093
λ=0.15	0.918	0.779	0.755	0.788	0.959
λ=0.20	0.912	0.754	0.708	0.723	0.866
λ=0.25	0.908	0.732	0.669	0.678	0.808
λ=0.30	0.904	0.714	0.633	0.642	0.756
	r2pred>=0.7				
λ=0.05	0.938	0.861	0.889	0.994	1.209
λ=0.10	0.929	0.816	0.814	0.864	1.093
λ=0.15	0.938	0.820	0.778	0.793	0.965
λ=0.20	0.932	0.794	0.731	0.729	0.871
λ=0.25	0.931	0.774	0.692	0.685	0.813
λ=0.30	0.930	0.757	0.657	0.651	0.760
	r2pred>=0.8				
λ=0.05	0.956	0.903	0.916	0.997	1.211
λ=0.10	0.952	0.859	0.841	0.869	1.094
λ=0.15	0.950	0.831	0.790	0.801	0.970
λ=0.20	0.947	0.808	0.746	0.742	0.887
λ=0.25	0.943	0.786	0.704	0.693	0.814
λ=0.30	0.939	0.767	0.665	0.654	0.771
	r2pred>=0.9				
λ=0.05	0.970	0.916	0.926	0.985	1.200
λ=0.10	0.965	0.875	0.855	0.835	1.068
λ=0.15	0.959	0.838	0.770	0.780	0.934
λ=0.20	0.959	0.829	0.746	0.767	0.913
λ=0.25	0.957	0.819	0.730	0.753	0.893
λ=0.30	0.956	0.811	0.705	0.743	0.938

Supplementary Table 2| Ratio of the observed number of significant associations in DISH results to SNP2HLA results at various lambda values, association *P* threshold levels, and *r2pred* values in the European null-model dataset.

	Association *P* threshold				
	0.5	0.05	0.005	0.0005	0.00005
	r2pred>=0				
λ=0.05	0.923	0.841	0.918	1.249	2.113
λ=0.10	0.904	0.775	0.780	0.966	1.403
λ=0.15	0.890	0.727	0.691	0.781	1.023
λ=0.20	0.879	0.689	0.624	0.652	0.795
λ=0.25	0.868	0.656	0.571	0.562	0.666
λ=0.30	0.859	0.627	0.527	0.494	0.548
	r2pred>=0.5				
λ=0.05	0.931	0.847	0.922	1.253	2.115
λ=0.10	0.914	0.782	0.785	0.971	1.405
λ=0.15	0.903	0.738	0.698	0.787	1.028
λ=0.20	0.897	0.705	0.635	0.663	0.804
λ=0.25	0.888	0.673	0.582	0.572	0.673
λ=0.30	0.881	0.645	0.537	0.502	0.550
	r2pred>=0.6				
λ=0.05	0.935	0.853	0.928	1.260	2.130
λ=0.10	0.923	0.794	0.795	0.976	1.401
λ=0.15	0.913	0.748	0.696	0.760	0.951
λ=0.20	0.905	0.712	0.632	0.642	0.752
λ=0.25	0.899	0.684	0.583	0.561	0.641
λ=0.30	0.894	0.659	0.542	0.496	0.529
	r2pred>=0.7				
λ=0.05	0.944	0.862	0.916	1.183	1.829
λ=0.10	0.933	0.805	0.789	0.933	1.259
λ=0.15	0.925	0.765	0.706	0.760	0.935
λ=0.20	0.920	0.736	0.647	0.649	0.764
λ=0.25	0.919	0.709	0.596	0.566	0.643
λ=0.30	0.912	0.681	0.550	0.489	0.482
	r2pred>=0.8				
λ=0.05	0.954	0.879	0.921	1.165	1.761
λ=0.10	0.950	0.828	0.783	0.884	1.119
λ=0.15	0.941	0.784	0.693	0.690	0.726
λ=0.20	0.938	0.759	0.641	0.591	0.549
λ=0.25	0.936	0.740	0.606	0.536	0.490
λ=0.30	0.932	0.720	0.574	0.488	0.408
	r2pred>=0.9				
λ=0.05	0.974	0.904	0.880	0.968	1.097
λ=0.10	0.967	0.860	0.779	0.806	0.800
λ=0.15	0.960	0.823	0.710	0.669	0.625
λ=0.20	0.956	0.802	0.673	0.614	0.548
λ=0.25	0.953	0.787	0.645	0.569	0.501
λ=0.30	0.949	0.768	0.615	0.528	0.447
